# Erythropoietin and Non-Erythropoietic Derivatives in Cognition

**DOI:** 10.3389/fphar.2021.728725

**Published:** 2021-09-06

**Authors:** Samuel S. Newton, Monica Sathyanesan

**Affiliations:** ^1^ Division of Basic Biomedical Sciences, Sanford School of Medicine, University of South Dakota, Vermillion, SD, United States; ^2^ Sioux Falls VA Healthcare System, Sioux Falls, SD, United States

**Keywords:** neurotrophic, erythropoietin receptor, synaptic plasticity, hippocampus, neurogenesis

## Abstract

Cognitive deficits are widespread in psychiatric disorders, including major depression and schizophrenia. These deficits are known to contribute significantly to the accompanying functional impairment. Progress in the development of targeted treatments of cognitive deficits has been limited and there exists a major unmet need to develop more efficacious treatments. Erythropoietin (Epo) has shown promising procognitive effects in psychiatric disorders, providing support for a neurotrophic drug development approach. Several preclinical studies with non-erythropoietic derivatives have demonstrated that the modulation of behavior is independent of erythropoiesis. In this review, we examine the molecular, cellular and cognitive actions of Epo and non-erythropoietic molecular derivatives by focusing on their neurotrophic, synaptic, myelin plasticity, anti-inflammatory and neurogenic mechanisms in the brain. We also discuss the role of receptor signaling in Epo and non-erythropoietic EPO-mimetic molecules in their procognitive effects.

## Introduction

The prevalence of cognitive dysfunction, which includes deficits in any of the broad domains of attention, learning and memory, language, perception and executive function, is widespread in neuropsychiatric disorders (Millan et al., 2012). Cognitive dysfunction is a central element of schizophrenia and appears to precede the expression of positive symptoms (Marder et al., 2004; Bowie and Harvey, 2006). Antipsychotics are effective in treating psychosis but only marginally improve cognition, with second generation antipsychotics showing better efficacy than first generation drugs (Hill et al., 2010). A parallel can be seen in major depression, where cognitive dysfunction is a key factor in disease associated psychosocial and functional impairment. The incidence of cognitive deficits is particularly high in late-life depression and in women (Yaffe et al., 1999; Koenig et al., 2014). Antidepressants, which are widely prescribed to improve mood, have limited efficacy in ameliorating cognitive deficits. A large clinical study involving over 1,000 patients tested three extensively prescribed antidepressants, escitalopram, sertraline and venlafaxine, and found no improvement in any of the nine cognitive measures examined (Shilyansky et al., 2016). Vortioxetine, a SSRI antidepressant which has shown specific improvement in cognitive function in major depression in comparison to other antidepressants, does so independent of depressive symptoms (McIntyre et al., 2014; Baune et al., 2018; Vieta et al., 2018). Whether cognitive deficits are a frequent but distinct entity in neuropsychiatric disorders or if they are coupled to specific diseases is worthy of debate (Millan et al., 2012). The debilitating consequences of cognitive dysfunction is however beyond debate and has emerged as a major unmet need in the field. The acute regulation of neurotransmitter levels, predominantly acetylcholine, as seen in prescription Alzheimer’s disease drugs, is the current approach in the field to ameliorate cognitive impairment. The use of acetylcholinesterase inhibitors in AD is based on the cholinergic hypothesis, which implicates a deficit in acetylcholine for memory impairment (Bartus et al., 1982). However, AD is a multifactorial disease and cholinergic drugs have shown only marginal efficacy, are accompanied by adverse side effects and incapable of halting or slowing cognitive decline (Russ and Morling, 2012; Sharma, 2019) The limitations of this mechanism of action is evident in the modest short-term improvement in cognition that progressively dissipates with disease progression (Hogan, 2014; Blanco-Silvente et al., 2017). In this review, we consider the clinical and preclinical evidence in favor of a neuroprotective and neurotrophic mechanism of action molecule, erythropoietin and its non-erythropoietic derivatives, to better understand and treat cognitive dysfunction. Studies discussed here are from focused PubMed searches, our work and knowledge of the field. We start with a discussion of results from clinical studies and then examine evidence from preclinical investigations to shed light on molecular and cellular mechanistic aspects.

## Review

### Clinical Studies of Erythropoietin

Promising results from clinical testing of erythropoietin (EPO) in multiple psychiatric diseases (Table 1) strengthen the hypothesis that it produces behavioral effects via central mechanisms associated with the neurobiology of cognition. Seminal studies demonstrating efficient transport of EPO across the blood brain barrier in humans set the stage for further clinical testing in different disease settings (Ehrenreich et al., 2004). EPO appears to be a promising candidate for chronic progressive multiple sclerosis as evidenced by the results of an open-label study reporting reductions in the disability status scale and cognitive symptoms (Ehrenreich et al., 2007a). As an add-on drug in chronic schizophrenia, EPO significantly improved cognitive function over placebo and reduced levels of glial damage marker S100B (Ehrenreich et al., 2007b). Interestingly, EPO appears to be the only drug capable of delaying the progressive thinning of the cortex that occurs in schizophrenia, correlating with an improvement in attention and memory (Wustenberg et al., 2011). Functional magnetic resonance imaging (fMRI) analysis of healthy volunteers showed that a single dose of EPO increased bilateral hippocampal blood oxygenation level-dependent (BOLD) signal during picture encoding tasks (Miskowiak et al., 2007b). The subjects were imaged 1 week after the acute dose and hematocrit was unchanged, demonstrating that the hippocampal effects of EPO are independent of erythropoiesis. It is therefore likely that mechanisms such as modulation of synaptic plasticity are involved in EPO’s acute hippocampal actions. The cognitive effects of EPO are discernible at 3 days after an acute dose, based on enhanced recognition of facial expressions (Miskowiak et al., 2008). However, EPO’s effect on spatial working memory and verbal fluency were significant only at 7 days but not 3 days after administration, indicating that certain cognitive measures are differentially influenced over time (Miskowiak et al., 2007a). An extended dosing regimen, 8 weeks of 40,000 IU/week, in remitted bipolar disorder patients, improved attention, facial recognition and processing speed but had no effect on verbal memory (Miskowiak et al., 2014a). It is noteworthy that the improvements in sustained attention and executive function were realized 6 weeks after the treatment regimen. This suggests the involvement of cellular phenomena such as hippocampal neurogenesis in the delayed expression of particular cognitive effects.

**TABLE 1:** Clinical studies of the cognitive effects of EPO in psychiatric diseases.

Psychiatric disease	Dosing regimen	Subjects	Tests	Results	References
Schizophrenia	40,000 IU/wk 3 months	39	RBANS, WCST-64	Improved RBANS and WCST-64 No change in psychopathology, social functioning	Ehrenreich et al., Mol Psych:1–15, 2006
Schizophrenia	40,000 IU/wk 3 months	32	RBANS	Halted gray matter loss in the right ventral frontal cortex, ventral striatum and left entorhinal cortex. Improved memory and attention No change in psychopathology	Wustenberg et al., Mol Psych:1–11, 2010
Normal volunteers	40,000 IU - single dose	23	Picture encoding recognition memory	Increased hippocampal activation	Miskowiak et al. (2007b)
Normal volunteers	40,000 IU - single dose	24	Facial recognition self reported mood	Improved facial recognition and mood	Miskowiak et al. (2007a)
Bipolar Disorder	40,000 IU/wk 8 weeks	43	RAVLT, RBANS, RVP WAIS III	No change in verbal fluency Improved attention, processing speed and facial recognition	Miskowiak et al. (2014b)
Major Depression	40,000 IU/wk 8 weeks	31	HDRS-17, GAF, RAVLT BDI-21, WHOQOL-BREF	No change in HDRS-17. Improvement in and verbal memory	Miskowiak et al., Neuropsychopharm 1,399–1,408, 2010
Unipolar Depression Bipolar Disorder	40,000 IU/wk 8 weeks	69	RAVLT	Mood-independent improvement in verbal	Miskowiak et al. (2015)

Clinical studies of erythropoietin in cognition. Shown in the table are details of study populations, dosing regimen employed, number of subjects, tests, results and references. RBANS–Repeatable Battery for the Assessment of Neuropsychological Status, WCST–Wisconsin Card Sorting Test, RAVLT–Rey Auditory Verbal Learning Test, RVP–Rapid Visual Information Processing, WAIS III–Wechsler Adult Intelligence Scale 3^rd^ edition, HDRS–Hamilton Depression Rating Scale, GAF–Global Assessment of Functioning, BDI–Beck Depression Inventory and WHOQOL-BREF - World Health Organization Quality of Life–Abbreviated.

In a randomized, double-blind, placebo-controlled study of treatment-resistant depression, EPO did not improve Hamilton Depression Rating Scale-17 (HDRS-17) scores after an 8-weeks dosing regimen (Miskowiak et al., 2014b). However, there was significant improvement in the Beck’s Depression Inventory-21 (BDI-21), World Health Organization Quality of Life-BREF (WHOQOL-BREF) score and mood-independent verbal memory, that were maintained for at least 6 weeks after the last dose of EPO (Miskowiak et al., 2014b). The persistence of the cognitive gains indicates that the effects are likely independent from hematocrit regulation. Whether the study was potentially under-powered to detect an effect in HDRS-17 or EPO has a more pronounced effect in cognitive domains is worthy of further discussion and study. It is also important to note that although the increase in hematocrit normalized 2 weeks after treatment cessation, a role for the altered hematological parameters, albeit earlier during treatment, in the observed effects cannot be dismissed. Post-hoc analysis of EPO’s effects in bipolar and unipolar disorder revealed that the speed of complex cognitive processing was significantly improved across affective disorders, with the effects continuing for at least 6 weeks after the last dose of treatment (Ott et al., 2016; Petersen et al., 2018). MRI analyses indicate that the mood-independent improvement in memory observed in depression correlates with reversal of left hippocampal volume loss, specifically in the CA1-CA3 and subiculum (Miskowiak et al., 2015). The effects indicate a combination of neuroprotective and neurotrophic actions, halting or slowing shrinkage and recovering volumetric loss. Understanding precisely how this morphological and structural recovery occurs and which cell types are involved will require focused preclinical investigations. The specificity of EPO’s action on the left hippocampus is interesting as previous evidence in major depression shows larger volume loss in the left hippocampus (Roddy et al., 2019). This is somewhat reminiscent of the findings in schizophrenia where EPO halted the loss of gray matter in the cortex (Wustenberg et al., 2011). Could it be that the regenerative nature of EPO’s cellular actions are most prominent in brain regions and subregions that exhibit pronounced consequences of disease pathophysiology? From a therapeutic standpoint, it will be important to test the relationship between volumetric recovery and the alleviation of disease symptoms.

### Molecular Mechanism of Action

The results from clinical studies suggest that while EPO can produce cognitive effects as early as 3 days with a single dose, continued dosing for 8 weeks improves efficacy (Miskowiak et al., 2008; Miskowiak et al., 2014b). The beneficial effects are maintained for at least 2 months or more after therapy cessation (Miskowiak et al., 2015). The early onset of therapeutic effects contrasts with currently prescribed antidepressant and antipsychotic drugs which exhibit a latency of a few weeks to a month to produce therapeutic effects (Machado-Vieira et al., 2010). A potential explanation is that the behavioral effects of prescription antidepressants are dependent on the activation of neurotrophic signaling, which requires chronic dosing, whereas direct delivery of a neurotrophic molecule produces faster effects (Duman et al., 1997; Shirayama et al., 2002). As an FDA-approved drug with a strong safety profile, EPO can be used in the clinic to experimentally test hypotheses regarding the role of neuroprotective, neurotrophic and neurogenic actions to alleviate disease-induced dysfunction. Furthermore, because it has been used extensively in the clinic, useful data from associative correlation studies involving large patient populations is available (Hung et al., 2019). In taking this approach it will be important to consider two questions. What molecular mechanisms are involved in EPO’s behavioral effects? Can they enhance our understanding of cognitive deficits in neuropsychiatric diseases?

### Insight From Gene Expression Analysis

Transcriptomic methods examining EPO-induced gene regulation in neuronal cells and brain tissue have yielded useful information to develop mechanism of action understanding. In neuronal phenotype PC12 cells, EPO (25 nM) elicited maximal gene regulation at 3 h and returned to baseline at 6 h (Renzi et al., 2002). However, a low (10 pM) concentration of EPO required a long exposure for 24 h toincrease the anti-apoptotic gene Bcl-xl and strikingly decreased the pro-apoptotic gene Bak, indicating that EPO produces differential time and concentration-dependent effects (Renzi et al., 2002). Neurotrophic factor genes such as brain derived neurotrophic factor (BDNF), VGF (non-acronymic) and neuritin are elevated by EPO and the non-erythropoietic derivative, carbamoylated EPO (CEPO), in the rat hippocampus (Girgenti et al., 2009; Sathyanesan et al., 2018), and specifically in the dentate gyrus (Tiwari et al., 2019). These genes are also induced by physical exercise (Hunsberger et al., 2007) and have been independently shown to improve cognitive function (Blurton-Jones et al., 2009; Choi et al., 2014; Lin et al., 2015). The increase in BNDF along with other immediate early genes such as Arc, Egr 1 and Fos suggests that EPO’s cognitive effects could occur via modulation of synaptic plasticity as these molecules are known to participate in mechanisms essential for learning and memory (Cunha et al., 2010; Minatohara et al., 2015). An unbiased genome-wide transcriptomics study of CEPO-induced gene regulation, followed by bioinformatics analysis, provides additional support for the hypothesis that EPO’s actions in the brain occur by a neurotrophic and synaptic plasticity mechanism (Tiwari et al., 2019). Interestingly, the data showed c-AMP regulated binding protein (CREB)-signaling to be a significantly activated pathway, and phospho-CREB was shown to be upregulated in the dentate gyrus (Sampath et al., 2020). CREB is a heavily studied transcription factor in psychiatric neuroscience, encompassing key roles in diverse areas, including drug addiction (Carlezon et al., 1998), antidepressant activity (Newton et al., 2002) and memory (Silva et al., 1998). The fact that CREB is a crucial mediator of neuronal neurotrophic activity (Finkbeiner et al., 1997) and a modulator of processes involved in memory formation (Silva et al., 1998; Tully et al., 2003) makes it a potential hub molecule that can link EPO’s neurotrophic activity with cognitive effects. The induction of CREB by EPO is not directly indicated by the canonical EPO signal transduction cascade (Richmond et al., 2005) and points to the usefulness of unbiased, global methods to identify novel pathways. While EPO-induced gene regulation data in neural tissue is not extensive, available evidence allows comparison of gene regulatory changes in different tissues and brain regions by EPO and non-erythropoietic EPO-mimetic molecules. The precise role played by the regulated molecules in EPO’s cognitive effects can be further tested by transgenic gene knockout/overexpression, *in vivo* gene manipulation and pharmacological experiments.

### Cellular Actions of Erythropoietin

Gene expression and signal transduction studies yield information on the acute effects of EPO but are insufficient in understanding sustained therapeutic benefits. For example, the regulation of neurotrophic genes after a short course of EPO can implicate trophic support and synaptic mechanisms in the improvement in mood and/or cognitive function. However, examining the cell-level consequences of gene and protein regulation is necessary to understand how the morphological, structural and behavioral changes are maintained for months after drug administration. It is in this context that the synaptic and neurogenic actions of EPO become important.

### Synaptic Plasticity

The key and essential role played by neurotrophic factors in synaptic plasticity and synapse formation is well established (Vicario-Abejon et al., 2002). In addition, the strong relationship between synapse integrity and cognitive function leads to a viable mechanism whereby EPO exerts cognitive effects via neurotrophic signaling. Long-term potentiation (LTP), a central mechanism for learning, is increased by EPO in the CA1 of the hippocampus (Adamcio et al., 2008). Administration of 11 but not 3 doses improved hippocampal-dependent memory that persisted for another 3 weeks after the end of the dosing regimen. Electrophysiological recordings revealed a dual and demarcated mechanism of action, silencing of a substantial group of synapses but an excitation of certain neuronal circuits (Adamcio et al., 2008). This dual action is likely to be important in disease conditions to restore the balance between excitatory and inhibitory transmission. Differential synaptic effects were observed in the hippocampus with acute (40–60 min) vs extended (3 h) exposure to EPO (Dias et al., 2018). While acute EPO inhibited excitatory synaptic transmission, extended exposure followed by a recovery period (1 h) produced a non-canonical AMPA receptor-dependent increase in transmission (Dias et al., 2018).

The LTP-enhancing effect of EPO is particularly pertinent in diseases with prominent cognitive dysfunction such as Alzheimer’s disease (AD), where it rescues impaired LTP and memory deficits (Esmaeili Tazangi et al., 2015). To develop EPO or non-erythropoietic mimetics as procognitive drugs it will be important to understand the mechanistic relationship between the dosing regimen, cellular consequences and time course of therapeutic effects. A high dose (5000 IU/kg) of EPO, delivered on alternate days for 3 weeks, and tested in the 5-choice serial reaction time test, showed that the cognitive improvement was maintained for 3 months (El-Kordi et al., 2009). Due to the young age of the animals (postnatal day 28), the lasting effects could be due to EPO influencing neural networks and gray matter volume at this stage of development (Semple et al., 2013). Specific neurodevelopmental actions of EPO on the early hippocampal network are emerging. Recently, EPO was shown to drive the maturation of the GABAergic system in the postnatal mouse hippocampus by using a constitutively expressing transgenic mouse line (Khalid et al., 2021). The actions on the GABAergic interneurons appear to be indirect as the EPO receptors (EPOR) are expressed on pyramidal cells. However, multiple aspects of the GABAergic network were regulated, including increased expression of GABAergic cells and synapses, faster maturation and reduced early postnatal apoptosis in the CA1 and CA3 due to EPO’s well known anti-apoptotic properties (Khalid et al., 2021). EPO’s acute and extended synaptic plasticity effects and its ability to modulate both excitatory and inhibitory neurotransmission can in part contribute to the observed cognitive behavioral effects after a single administration and continued improvement with additional dosing.

### Erythropoietin-Induced Oligodendrogenesis

The recent interest in understanding brain function in health and disease from a brain circuitry angle, has made it important to focus attention on an understudied cell type in the brain, oligodendrocytes. These myelin-forming cells play key roles in the neural circuits associated with cognitive function. The formation of oligodendrocytes from oligodendrocyte precursor cells, oligodendrogenesis, is dynamically regulated by neural activity (Hughes et al., 2018; Mitew et al., 2018). The importance of this relationship was recently demonstrated by studies showing that inhibition of adult oligodendrogenesis in hippocampal-cortical circuits resulted in impaired spatial memory and fear memory consolidation (Steadman et al., 2020). As no changes were observed in myelin patterns, the effects appear to be a result of subtle modulation of myelin plasticity. These results are pertinent to EPO’s mechanism of action in several brain injury models. The heightened vulnerability of neonatal brain white matter (WM) to ischemic insult is well known and results in irreversible deficits. EPO administered 24 h after neonatal hypoxia/ischemia (H/I) increased both neurogenesis and oligodendrogenesis and improved neurological parameters despite having no impact on infarct size (Iwai et al., 2010). It is interesting to note that EPO’s repair of WM and concomitant effects on sensorimotor behavior were observed only after 14 days, indicating that Epo-induced myelination occurs slowly over time (Iwai et al., 2010). The long-term impact of the pro-myelinating effects of EPO were shown in an early postnatal hyperoxia model where drug administration at P3-P6 improved cognition in adolescent and adult rats (Dewan et al., 2020). The latency associated with the WM cell level actions of EPO and the long-lasting impact on cognitive function are intriguing. It could be that recovery of WM injury during neurodevelopment is a slow process, but once achieved, the results are persistent because it involves repair at the level of brain circuits. Insight into the molecular mechanism of action obtained by microarray analysis of an EPO-treated oligodendrocyte precursor cell line show the induction of several lipid transport and lipid metabolism genes in addition to pro-myelinating trophic factors, Igf1 and Igf2 (Gyetvai et al., 2017). As these results were obtained in a cell line that required overexpression of EPOR, it will be important to test whether EPO produces a comparable gene profile *in vivo* at physiological levels of EPOR expression. Examining EPO’s effects on myelin plasticity in the absence of injury will also be instructive to the neurocognition field.

### Erythropoietin and Anti-Inflammatory Mechanisms

An area of investigation that is attracting significant recent attention from both neurodegenerative and psychiatric illnesses is inflammation. Revision of erroneous dogma that characterized the brain as an immune-privileged organ has opened wide this field of research and resulted in the identification of CNS cells, molecules and markers associated with inflammation. Chronic neuroinflammation is linked with cognitive decline in aging (Ownby, 2010)and the neurobiology of depression (Miller and Raison, 2016). Cytokines such as IL-1β, IL-6 and TNF-α have emerged as key immune response molecules. As the knowledge base of inflammation as an important contributing factor to CNS diseases expanded, it led to research efforts aimed at identifying anti-inflammatory therapeutic molecules. EPO emerged as a viable candidate molecule with several studies reporting anti-inflammatory properties in diverse animal models. EPO was shown to produce an anti-inflammatory effect in a rat model of experimental autoimmune encephalomyelitis (EAE), reducing levels of IL-6 and delaying the release of TNF in the spinal cord (Agnello et al., 2002). As microglia are important mediators of the inflammation response in the brain it is interesting to note that non-erythropoietic CEPO showed anti-inflammatory properties by reducing microglial activation and rescuing neurological deficits in a focal cerebral ischemia stroke model (Villa et al., 2007). Lipopolysaccharide (LPS), an endotoxin, that is frequently used as an agent to evoke a powerful immune response, caused cognitive impairment and elevated IL-1β, which was resolved by EPO (Gao et al., 2015). Despite the promising results, understanding the precise mechanistic relationship between EPO and inflammation has been somewhat elusive. EPO-induced cognitive improvement in depressed patients was not associated with changes in plasma levels of pro-inflammatory markers such as IL-6, IL-18 and high sensitive c-reactive protein (Vinberg et al., 2016). In an *in vitro* study, EPO showed no anti-inflammatory effects on microglia, neither suppressing release of TNF-α, nor reducing LPS-induced nuclear translocation of the pro-inflammatory transcription factor NF-κB (Wilms et al., 2009). The *in vivo* anti-inflammatory actions of EPO are likely to be indirect and not due to direct antagonism of inflammatory mechanisms. This was shown in a cerebral artery ischemia study where EPO was anti-inflammatory only when there was neurodegeneration-induced inflammation and was primarily due to its anti-apoptotic activity on neurons rather than direct interference of inflammatory cytokines (Villa et al., 2003). These findings give us direction on the applications of EPO and enable us to choose the disease settings in which beneficial effects are likely to occur from an informed perspective.

### Erythropoietin-Induced Neurogenesis

The phenomenon of adult hippocampal neurogenesis has been heavily investigated in preclinical studies and linked to antidepressant activity (Santarelli et al., 2003) and cognition (Costa et al., 2015). Although the existence of the phenomenon in humans can be deemed somewhat controversial (Sorrells et al., 2021), EPO-induced neurogenesis can be highly consequential for modulation of neuroplasticity and sustained cognitive improvement. EPO was shown to transiently increase subgranular zone (SGZ) neural progenitor cells by 30% without impacting long-term survival of newborn cells (Ransome and Turnley, 2007). Whether the outcome on survival would be different in pathological conditions of neurogenic deficit or cell loss is worthy of further discussion. Interestingly, a 3-weeks administration of EPO to adolescent mice resulted in a 20% increase in CA1/CA3 pyramidal neurons and oligodendrocytes in the absence of conventional SGZ neurogenesis (Hassouna et al., 2016). A volumetric increase in the hippocampus was noted in adult mice that were dosed with a similar EPO regimen. This is somewhat reminiscent of the volumetric recovery in the CA1-CA3 of depressed patients (Miskowiak et al., 2015). The observation that the increase in neurons occurs in 3 weeks and without mitotic cell division indicates that a re-routing of cell fate from an existing pool of precursor cells must be involved (Hassouna et al., 2016). A question that arises in the context of these findings is whether cells in the CA1 subfield are particularly responsive to EPO, and why? EPOR expression in the CA1 is among the highest in the brain, and expression of both EPO and EPOR are further elevated by the complex running wheel (CRW) cognitive challenge paradigm (Wakhloo et al., 2020). This paradigm produces a mild functional hypoxia in CA1 pyramidal cells and is likely related to the higher sensitivity of CA1 neurons to oxygen levels (Butt et al., 2021). As a gene that is highly induced by hypoxia, EPO levels rise rapidly in the CA1 as a protective physiological response. This EPO then acts via EPOR on surrounding precursor cells, transitioning them to a neuronal phenotype and thereby eliciting proliferation-independent neurogenesis (Wakhloo et al., 2020). Due to the central role of EPOR in EPO-driven neurogenesis it will be interesting to investigate if non-erythropoietic EPO derivatives are also capable of increasing neurogenesis in a similar manner.

### Non-Erythropoietic Erythropoietin Derivatives

Even as promising results from clinical and preclinical studies steadily accrue, the primary limitation in translating the EPO findings into the development of a mainstream neurotrophic drug is its potent erythropoietic activity. The adverse hematological consequences are also an impediment to fully testing an extended, chronic dosing regimen to obtain maximal therapeutic efficacy. Researchers in the field noticed EPO’s key limitation early and have directed effort towards precluding the erythropoietic effects but retaining the neurotrophic and behavioral effects. A detailed discussion of all the known non-erythropoietic EPO mimetic molecules is beyond the scope of this review. We will therefore focus on non-erythropoietic molecules that have been derived from EPO or its structure and shown efficacy in cognitive assays. This includes chemical modifications and peptides.

**FIGURE 1:**
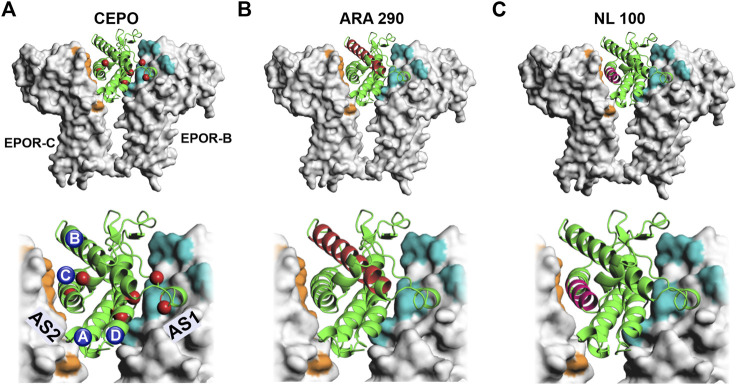
Non-erythropoietic EPO-mimetic molecules. EPO-mimetic molecules are shown superimposed onto the crystal structure of EPO bound to EPOR (PDB ID: 1EER) (Syed et al., 1998). In the top panel’s EPO (green cartoon) is shown bound to EPOR (gray) in molecular surface representation. High magnification images are shown in the bottom panels. Active site 1 is colored in teal and active site 2 in orange. **(A)** Carbamoylated EPO is shown bound to EPOR with carbamoylated residues represented by red spheres. The 4 helices are labeled alphabetically. **(B)** The residues that comprise the ARA 290 peptide from the B helix are shown in red (Brines et al., 2008). **(C)** The residues that comprise the NL-100 peptide from the C helix are shown in magenta (Dmytriyeva et al., 2019). AS 1- Active site 1, AS 2–Active site 2, EPOR-B–Erythropoietin receptor B chain, EPOR-C-Erythropoietin receptor C chain.

The discovery of carbamoylated EPO (CEPO) spawned a new direction in EPO neurobiology by demonstrating that the hematopoietic and neuroprotective actions of EPO can be dissociated (Leist et al., 2004). The *in vitro*, chemical addition of the carbamoyl group to specific amino acid residues (Figure 1A) renders EPO non-erythropoietic. The carbamoyl group consists of 4 atoms, one each of carbon, nitrogen, hydrogen and oxygen. Precisely how the addition of the 43 Da chemical moiety at seven residues (Sathyanesan et al., 2018) precludes activation of the erythropoietic signaling cascades is not fully understood. The modified sites are in proximity to both the high affinity active site 1 as well as the lower affinity active 2 of the EPO receptor dimers. Carbamoylation would likely negate salt bridges and alter ligand-receptor interaction at the modified residues. Receptor knockout studies indicate that CEPO binds to a heteroreceptor EPOR-betacommon receptor configuration rather than the conventional EPOR dimer (Brines et al., 2004). Preclinical studies have shown that CEPO rescues stress-induced cognitive deficits in rats and appear do so by neurotrophic actions that are predominantly in the dorsal hippocampus (Sathyanesan et al., 2018). A peptide derived from the B helix of EPO (Figure 1B) produced effects comparable to the AD drug galantamine in the object recognition test (Brines et al., 2008). As can be seen from the crystal structure and the location of the residues, the side chains would not be expected to interact with the receptor active sites. The effects could potentially be mediated by an alternate receptor. Interestingly, a 11-mer peptide consisting of adjacent residues facing the aqueous face of the helix also reproduced the therapeutic effects of the larger peptide, providing further support for the involvement of an alternate receptor (Brines et al., 2008). Another peptide, designed from the C helix of EPO (Figure 1C), improved spatial memory and reduced amyloid beta-induced neurotoxicity and memory deficits (Dmytriyeva et al., 2019). The effects were mediated via the EPOR despite the 12-mer peptide being potentially capable of interacting only with the low affinity active site 2 (Figure 1C). These findings raise important questions regarding the role of the receptor and the signaling pathway/s involved in the cognitive behavioral actions of EPO and non-erythropoietic EPO-mimetic molecules. Is there an intracellular signal transduction cascade that is responsible for the behavioral effects? And is that cascade activated by the EPOR-EPOR dimer as well as the EPOR-betacommon heteroreceptor?

### Erythropoietin Receptor Signaling

**FIGURE 2:**
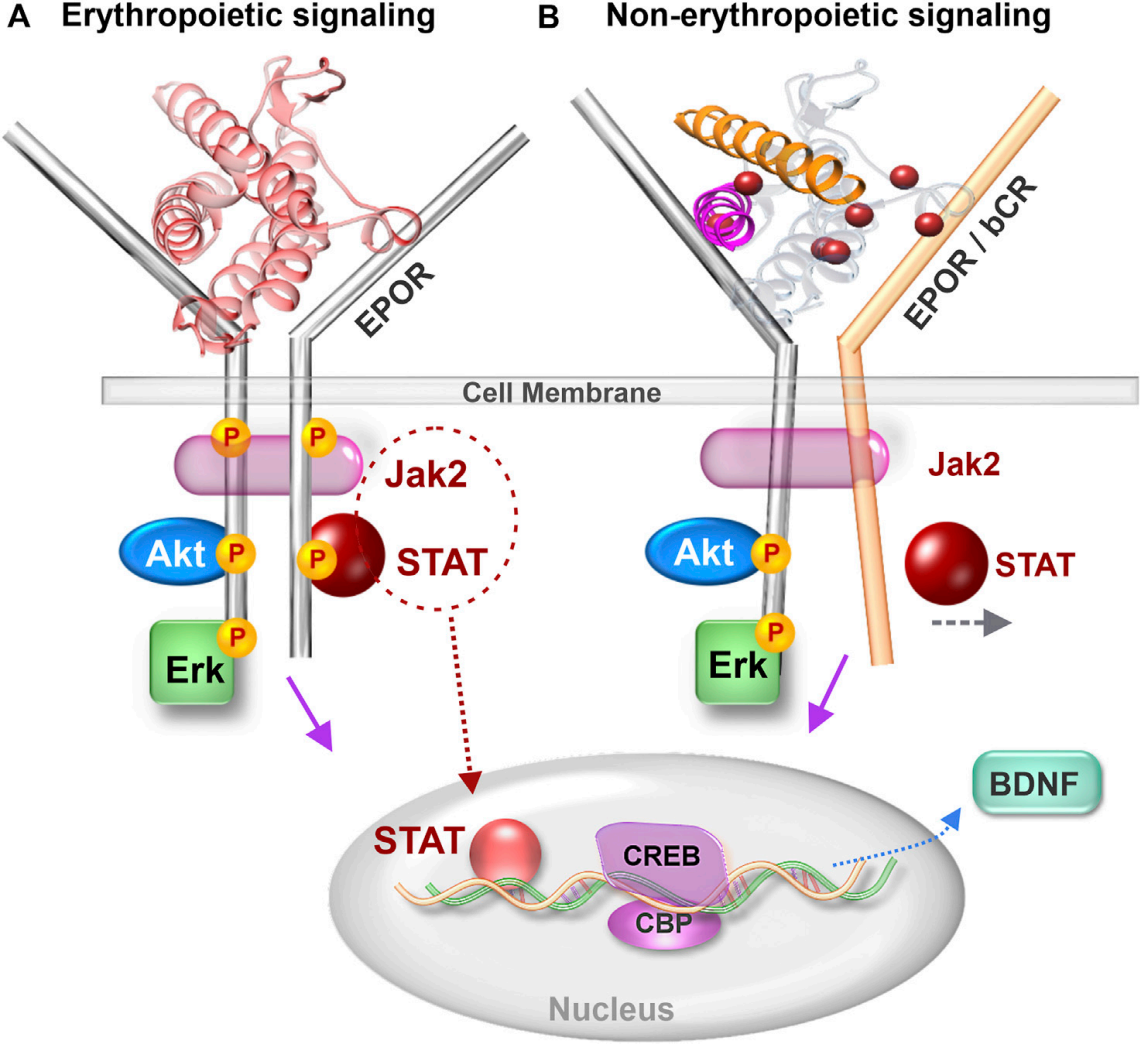
Comparison of erythropoietic and non-erythropoietic receptor signaling. **(A)** Erythropoietic signaling induced by EPO binding to EPOR dimer and causing Jak2 driven phosphorylation of STAT. **(B)** Non-erythropoietic signaling induced by binding of non-erythropoietic EPO-mimetic molecules such as CEPO, ARA 290 and NL 100. Non-erythropoietic molecules bind to either an EPOR-bCR heteroreceptor or cause the EPOR dimers to depart from the 120° angular orientation that supports erythropoiesis. Jak 2–Janus kinase 2, STAT - Signal transducer and activator of transcription, EPOR–erythropoietin receptor, bCR–betacommon receptor, CREB–cAMP response element binding protein, CBP–CREB binding protein and BDNF–Brain derived neurotrophic factor.

The JAK-STAT signaling cascade activated by EPO and transduced via the EPOR dimer is well studied in erythropoiesis (Richmond et al., 2005). The demonstration of functional EPOR expression in specific brain regions led to a new avenue of EPO signaling research in the CNS (Digicaylioglu et al., 1995). The significance of neuronal EPOR to cognitive performance was shown by transgenic mice expressing a constitutively active EPOR driven by the α-calcium/calmodulin-dependent protein kinase II promoter (Sargin et al., 2011). The superior cognitive capacities of these mice, albeit with slightly elevated impulsivity, established the importance of brain EPOR signaling in modulating behavior. The expanding role of EPOR signaling in brain function and disease states has spurred the field to better understand intracellular signal transduction cascades activated by EPO and non-erythropoietic derivatives. In addition to the erythropoietic JAK-STAT pathway (Digicaylioglu and Lipton, 2001), EPO also activates the PI3K-AKT and Erk signaling pathways in neurons (Siren et al., 2001). Further elucidation of the role of JAK-STAT signaling in EPO’s neuronal actions showed that STAT5 was essential for its neurotrophic effect but not for neuroprotection, indicating specificity of signaling pathways in relation to cellular effects (Byts et al., 2008). It is unlikely that the JAK-STAT cascade is activated by CEPO as neither JAK2 nor STAT5 are phosphorylated as a result of CEPO treatment (Leist et al., 2004). However, both AKT and ERK are phosphorylated by EPO and CEPO (Tiwari et al., 2021). While there is evidence for both AKT and ERK playing a role in cognitive function, the case for ERK signaling is much stronger (Peng et al., 2010). ERK signaling is essential for LTP (Schafe et al., 2008), synaptic plasticity (Thomas and Huganir, 2004) and neurotrophic activity (Ying et al., 2002). ERK also plays an important role in facilitating CREB-dependent gene transcription (Impey et al., 1998). Whether non-erythropoietic peptides such as ARA 290 and NL-100 induce ERK-signaling is currently unknown. The central role of EPOR in the actions of the parent peptide, Epotris, from which NL-100 (Figure 1C) was derived, has been clearly demonstrated (Pankratova et al., 2010). Although it binds EPOR with ∼10-fold lower affinity than EPO and STAT-5 phosphorylation is rather limited, the neurotrophic effects are lost upon knockout of EPOR and no association with the betacommon receptor was discernible (Pankratova et al., 2010). So how can the neuronal effects be mediated via EPOR and yet not cause an elevation in erythropoiesis? Perhaps this is a case of biased agonism, in which certain ligands stabilize the receptor in a conformation that supports neurotrophic but not erythropoietic signal transduction. The necessity of a 120° angular orientation between the receptor dimers for EPO-induced erythropoietic signaling has been shown (Syed et al., 1998). Even subtle departures from this critical orientation angle negatively influences JAK-STAT erythropoietic signaling (Wilson and Jolliffe, 1999). A viewpoint to consider isthat the other pathways are not as critically dependent on receptor orientation and continue to be transduced? We propose a model from that hypothetical angle (Figure 2) and caution that further work is necessary to test its validity.

A useful approach to investigate the potential for heteroreceptor involvement in cognitive behavior is to determine receptor colocalization in the cells that regulate the behavior of interest. For example, the neuroprotective role of EPO against seizure-induced hippocampal neurodegeneration was shown not to involve an EPOR-bCR heteroreceptor as bCR was expressed only in reactive microglia while EPOR was detected in neurons and astrocytes (Nadam et al., 2007). When bCR colocalized with EPOR, in the spinal cord and cerebellum, knockout of bCR resulted in the loss of EPO and CEPO’s protective effects in spinal cord injury (Brines et al., 2004). Colocalization studies highlight the importance of examining receptor expression in the context of the particular experimental model, tissue and cell types (Brines et al., 2004), because under physiological conditions brain EPOR expression correlates well with EPO expression but not with bCR (Sanchez et al., 2009).

An essential and necessary role for EPOR in EPO’s neurogenic and neuroplastic effects, leading to lasting cognitive improvement, has been demonstrated using elegant cell-type specific EPOR gene deletion experiments (Wakhloo et al., 2020). The currently available body of evidence suggests that EPOR and downstream signaling is required for EPO to produce cognitive effects. It is however unclear whether EPOR is a key component of a complex that includes bCR. A role for bCR independent of EPOR is unlikely because the bCR dimer does not independently bind cytokines (Broughton et al., 2015). Although an interaction between EPO and the EPOR-bCR heteroreceptor is possible based on computational structural biology modeling studies, rigorous biophysical analysis of the receptor extracellular domains indicated no association between EPOR and bCR, leaving open the possibility that potential associations could be mediated by the intracellular regions (Cheung Tung Shing et al., 2018). As the field progresses towards identifying the precise receptor configuration and/or confirmation responsible for the cognitive actions of EPO and non-erythropoietic mimetics, the possibility of an alternate receptor should not be ignored. Future research integrating receptor studies with sensitive, global signaling analyses such as phosphoproteomics methods can shed light on the subtle and dynamic intracellular changes that differentiate EPO-induced receptor signal transduction from that of non-erythropoietic analogs.
